# Identification of hepatitis B virus infection and integration and its oncogenic role in gastric cancer

**DOI:** 10.1002/ctm2.1601

**Published:** 2024-03-07

**Authors:** Mengge Li, Shusheng Wu, Jiayu Niu, Huiqin Luo, Wenju Chen, Lulu Cao, Ying Yan, Hong Tu, Yifu He

**Affiliations:** ^1^ Department of Medical Oncology The First Affiliated Hospital of USTC Division of Life Sciences and Medicine University of Science and Technology of China Hefei China; ^2^ State Key Laboratory of Systems Medicine for Cancer Shanghai Cancer Institute Renji Hospital Shanghai Jiao Tong University School of Medicine Shanghai China

Dear Editor,

Hepatitis B virus (HBV) infection has been considered an important role in hepatocellular carcinoma (HCC). However, the evidence between HBV and gastric cancer (GC) is limited.^1^ In this study, HBV integration is identified, for the first time, from HBV‐related GC and induces genomic instability and structural variations (SVs) in GC. HBV‐integrated genes may act as potential driver genes in GC carcinogenesis.

GC is an aggressive malignancy characterized by a high incidence and mortality globally.^2^ Growing evidence has shown an association between HBV infection and GC.^3^ However, the evidence is limited to sero‐epidemiological studies. Herein, twelve paired GC and para‐tumour tissues from patients with HBV surface antigen (HBsAg)‐seropositive who underwent radical resection were collected (Tables S1 and S2). HBV DNA (HBV S/C/P/X gene), especially covalently closed circular DNA (cccDNA), was important for HBV regulation and replication. By nested polymerase chain reaction (PCR),^4^ HBV S and/or C genes were detected in 7/12 of GC and 8/12 of para‐tumour tissues (Figure 1A and Table S2). Moreover, we used digital droplet PCR to determine the presence of cccDNA in the above HBV DNA‐positive tissues (Figure 1B). HBV cccDNA was detected in 85.7% of GC and 87.5 % of para‐tumour samples. To date, this is the first study to detect cccDNA in GC cells. However, there was no significant difference between the level of cccDNA in tumour and para‐tumour samples (Figure S1). We further performed a multiplex immunofluorescence assay of viral proteins in the above HBV DNA‐positive tissues (Figure 1C). Compared to that in para‐tumour tissues, the expression of HBcAg tended to increase in the GC tissues (Figure 1D). The expression of HBcAg (Figure 1E), rather than HBsAg or HBx expression (Figure S2), was significantly related to the level of cccDNA. The above data provide direct evidence demonstrating the HBV infection in GC.

**FIGURE 1:**
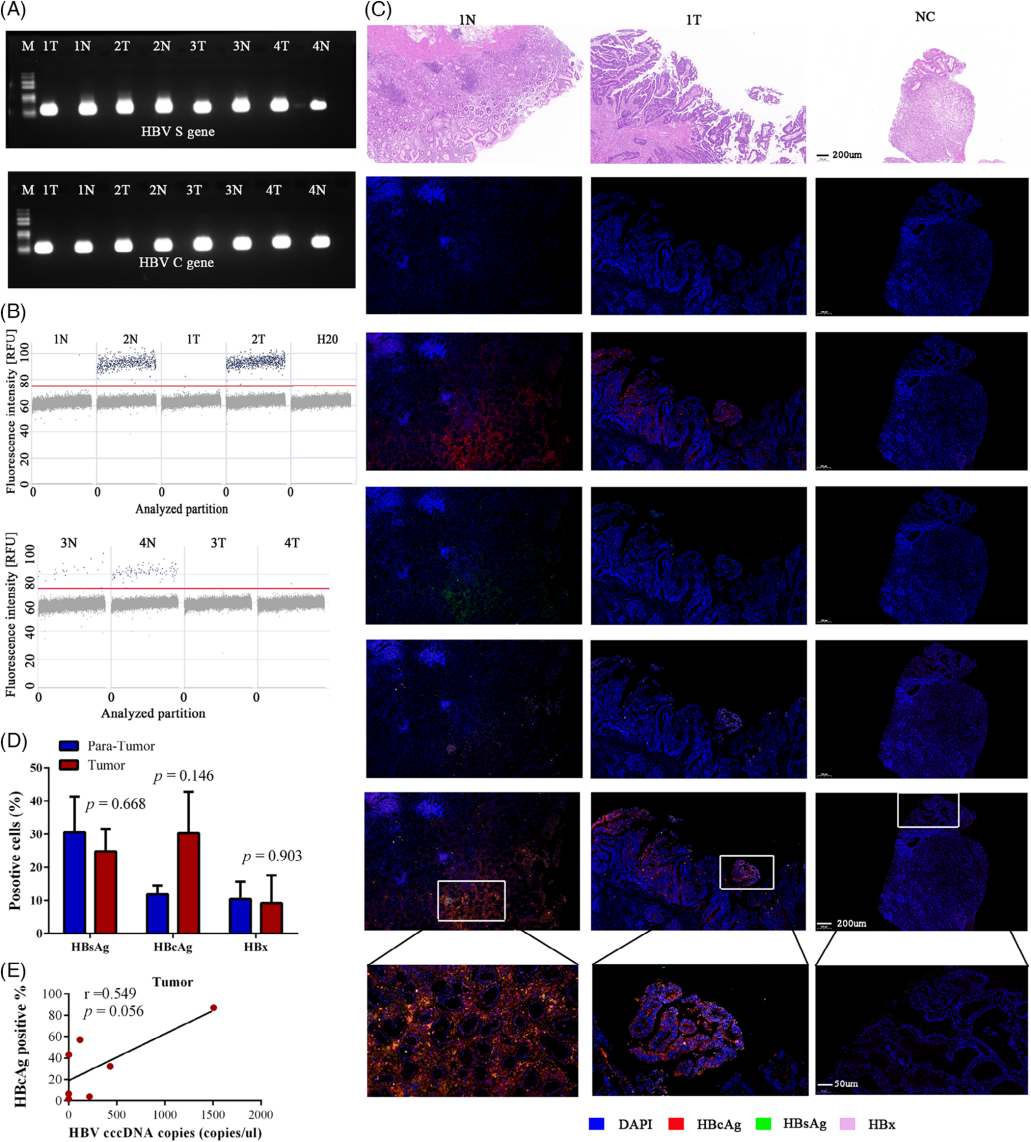
**Detection of hepatitis B virus (HBV) DNA, covalently closed circular DNA (cccDNA) and viral proteins in gastric cancer**. (A) Polymerase chain reaction amplification of the hepatitis B virus S gene and C gene in gastric cancer (GC) and para‐tumour tissues; (B) Amplification of HBV cccDNA by digital droplet polymerase chain reaction (ddPCR) in GC and para‐tumour tissues; (C). HBV surface antigen (HBsAg) (green), HBcAg (red) and HBx (pink) immunofluorescence staining representative of GC tissues and para‐tumour tissues from patient 1, NC, immunofluorescence staining representative of HBV negative tissues; (D) Percentage positive for HBsAg, HBcAg and HBx in GC tissues and para‐tumour tissues; (E) Correlation analysis between HBcAg and HBV cccDNA in GC tissues.

HBV DNA integration into the host genome has been considered an important mechanism for HBV‐related carcinogenesis in the liver^1^ and extrahepatic cancers.^4^, ^5^ To date, the role of HBV integration in GC has not yet been studied. Herein, the genomic DNAs of seven GC and eight para‐tumour tissues containing HBV S and/or C gene, and one paired sample (8 N and 8 T) that showed no existence of HBV DNA as control, were subjected to high‐throughput viral integration detection.^6^ A total of 434 integration sites (supporting read number ≥ 2) were identified from 71.4% (5/7) of HBV DNA‐positive GC and 87.5% (7/8) of para‐tumour tissues, including five paired tumour and para‐tumour samples (Tables S3 and S4). The discovery that HBV integrates into para‐tumour tissues may suggest its early occurrence post‐infection, potentially during the precancerous phase. Interestingly, we found that cccDNA levels were obviously related to the number of HBV integration sites (Figure 2A), indicating that the HBV integration events were correlated with viral replication.

**FIGURE 2:**
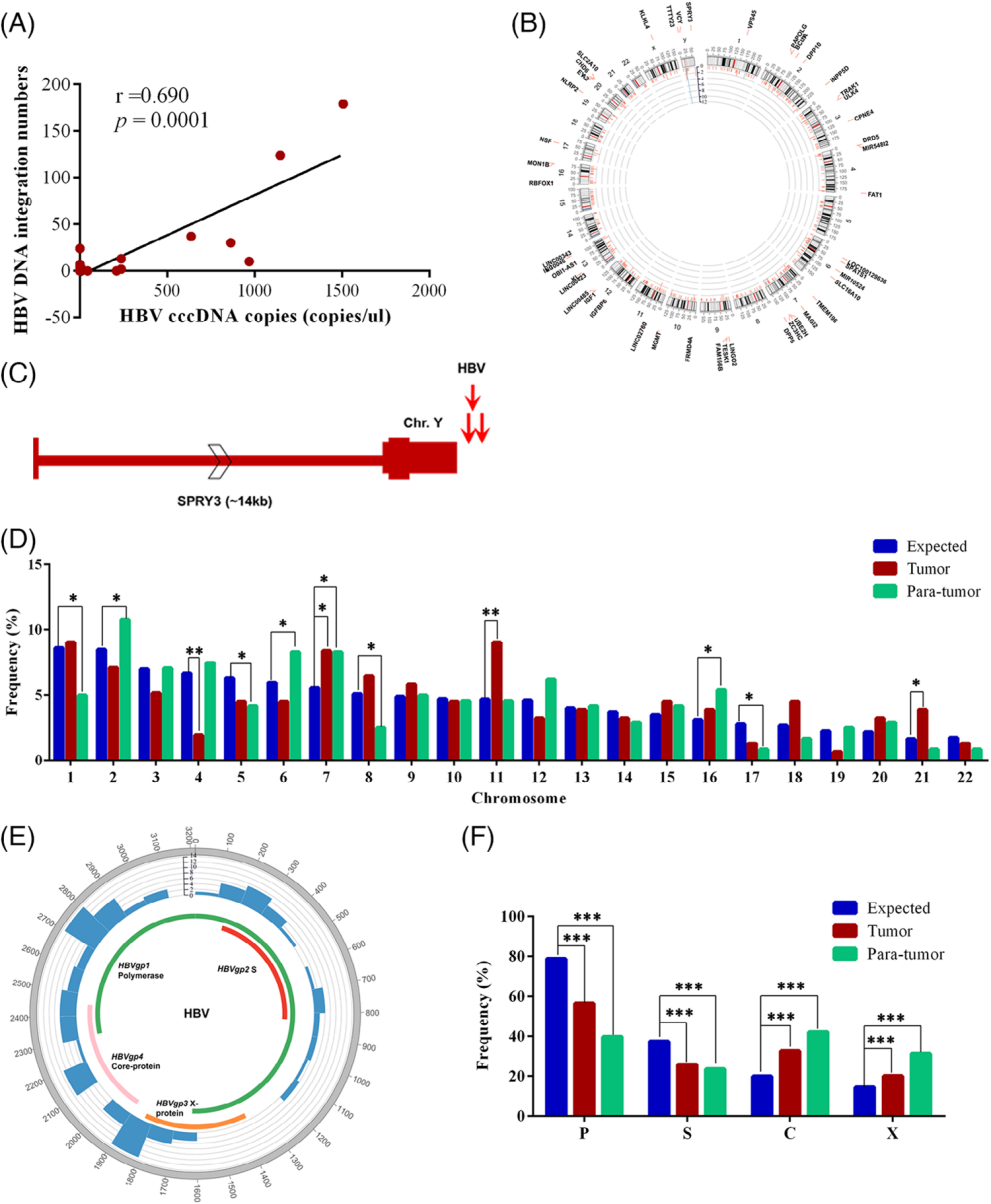
**Distribution of hepatitis B virus (HBV) DNA integration in viral and host genomes in gastric cancer**. (A) Analysis of the relationship between HBV covalently closed circular DNA (cccDNA) levels and HBV integration numbers; (B) Distribution of integration breakpoints in the host genome. Each bar represents the frequency of HBV DNA integration breakpoints at a particular locus in the human genome (hg19). Histogram axis units represent the number of breakpoints. Some loci with a high frequency of integration are marked; (C) HBV DNA repeatedly integrates into the gene sprouty RTK signaling antagonist 3 *(SPRY3)*. Red arrows represent the breakpoint sites of HBV DNA integration isolated from different tumour cell populations; (D) Chromosome enrichment of HBV DNA integration in the human genome. Each bar of whole‐chromosome represents the expected and the observed ratio of HBV DNA integration breakpoints at a particular chromosome in the human genome, A uniformly random distribution of breakpoints across the entire human genome or the viral genome was used to calculate the expected ratio of breakpoint number; (E) Distribution of integration breakpoints in the HBV genome. Histogram axis units represent the number of breakpoints. HBV genes with different functions are coloured; (F) Distribution of the observed (actual numbers) and expected (random distribution) ratio of breakpoints in HBV genetic elements. *p < .05; **p < .01; ***p < .001.

HBV integration sites occurred throughout the entire viral and human genomes, but some breakpoints were found in localized hotspots (Figure 2B,C). In GC samples, HBV integration was more frequently shown on chromosomes 7, 11 and 21 and occurred less frequently on chromosome 4 (Figure 2D). We also analyzed the integration breakpoint in the HBV genome. The viral‐host junctions were more prone to have occurred at the 3′‐end of the C gene and X gene both in tumour and para‐tumour tissues (Figure 2E,F). Besides, genomic instability‐correlated genomic elements around the HBV integration site were analyzed. We found that CpG islands were enriched (Figure S3), suggesting that HBV integration may increase genomic instability in GC.

To further investigate the role of viral integration on genomic instability and SVs, we performed a long‐read genome sequencing in two GC samples with the highest HBV integration events (2T and 2N, Figure 3A). The genome windows spanning HBV integration breakpoints within 500 kb were focused.^7^ Inversions were enriched around integration sites in both 2N and 2T samples (Figure 3B). Deletions near HBV integration breakpoints were larger in size than the other SVs in the 2T sample (Figure 3C), suggesting that integration events had a stronger genomic impact on these deletions. Moreover, the distance distribution of SVs to HBV‐integrated breakpoints was analyzed to explore the spatial connection between HBV integration and SVs. A raised proportion of deletions and insertions were observed close to the HBV integration sites (Figure 3D). Thus, the genomic instability and SVs induced by HBV integration may have significant functional effects on GC development, which requires further investigation.

**FIGURE 3:**
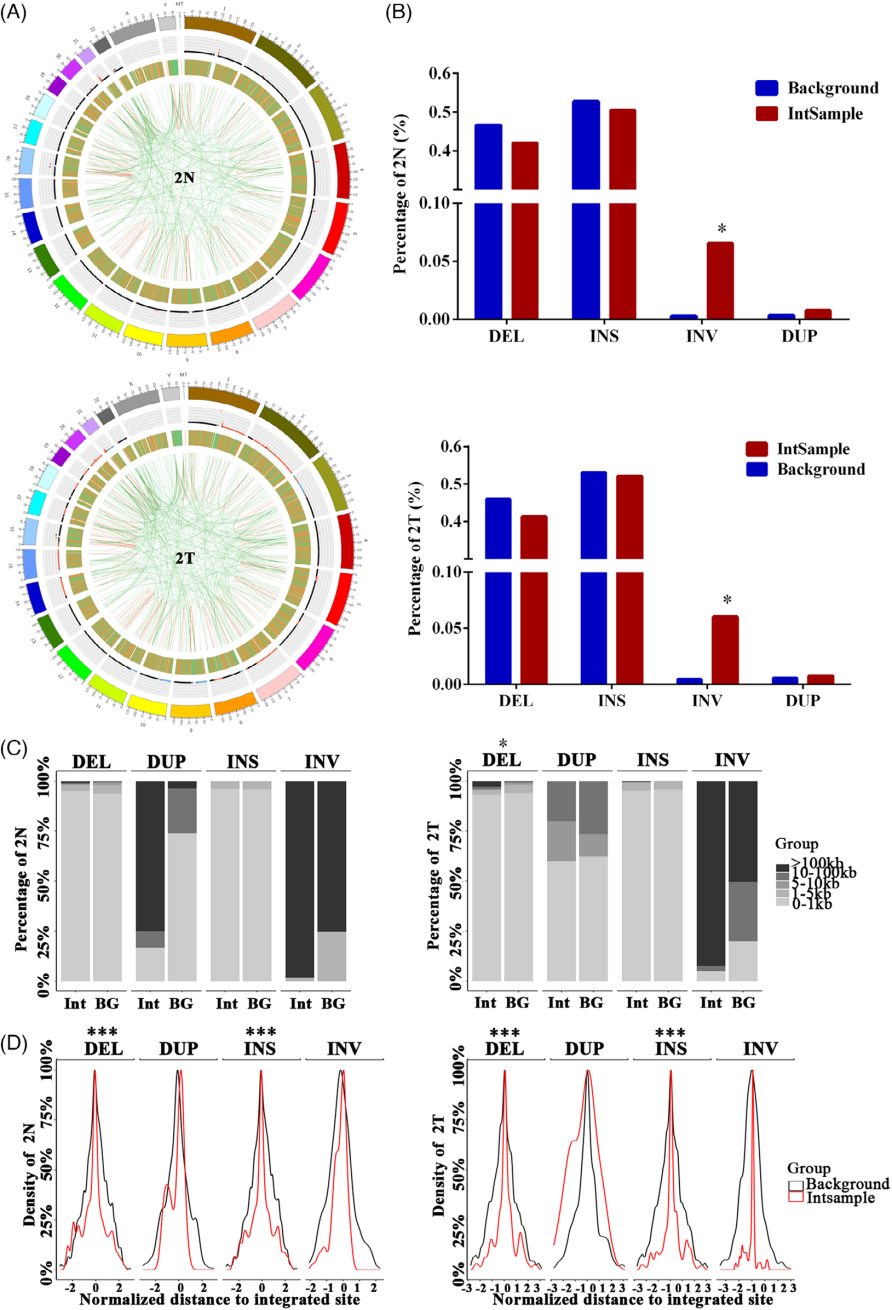
**The relationship between structural variations and hepatitis B virus (HBV) integration in gastric cancer**. (A) Distribution of integration breakpoints across human and virus genomes. The outermost circle is the chromosome information; the penultimate circle is the copy number, red represents gain/amplification, blue represents loss/deletion and grey represents normal copy number; the penultimate circle is the structural variation, green represents DEL, orange represents INS and red represents DUP; the innermost circle of lines represents translocation or inversion, the red line represents intrachromosomal translocation or inversion and the green line represents extrachromosomal translocation; (B) The relative proportion of structural variation (SV) types in different regions; (C) The SV size distribution of each SV type in different regions. (D) Distribution of normalized distance from SVs to nearest HBV integration sites. DEL, deletion; DUP, duplication; INS, insertion; INV, inversion; BG, background. *p < .05; **p < .01; ***p < .001.

Genes recurrently interrupted by HBV in HCC usually play a role in liver carcinogenesis.^8^ Moreover, cancer‐related genes with distant viral integrations can also be driven by HBV‐induced carcinogenesis.^9^ Recurrent HBV integration sites, defined as HBV targeting the same gene or inserting into the vicinal intergenic sequences within a 500 kb distance in different tissue samples are summarized in Table S5. Strikingly, sprouty RTK signalling antagonist 3 (*SPRY3*) was repeatedly inserted by HBV three times. *SPRY3* was repeatedly integrated by HBV 102 times in our previous analysis of viral integration in HCC tissues,^5^ suggesting that *SPRY3* may play some common and vital mechanisms in driving cancer progression. Then, we chose the *SPRY3* for further functional study. Consistent with the observation that *SPRY3* was obviously upregulated in GC tissues from the Cancer Genome Atlas (TCGA) dataset (Figure 4A), three GC cell lines exhibited higher *SPRY3* expression than the normal human gastric epithelial cell line at the mRNA level (Table S6 and Figure S4A). Clinicopathological analysis revealed that increased *SPRY3* was positively correlated with Lauren's classification and MSI status (Table S7). Patients with higher expression levels of *SPRY3* have shorter overall survival (Figure 4B), and *SPRY3* expression was an independent predictor of GC aggressiveness (Table S8). We then silenced *SPRY3* (Table S9 and Figure S4B) and found that the downregulation of *SPRY3* led to a significantly decreased rate of GC cell growth, migration and invasion (Figure 4C–E). Moreover, the silencing of *SPRY3* significantly decreased the tumour burden in the subcutaneous model (Figure 4F).

**FIGURE 4:**
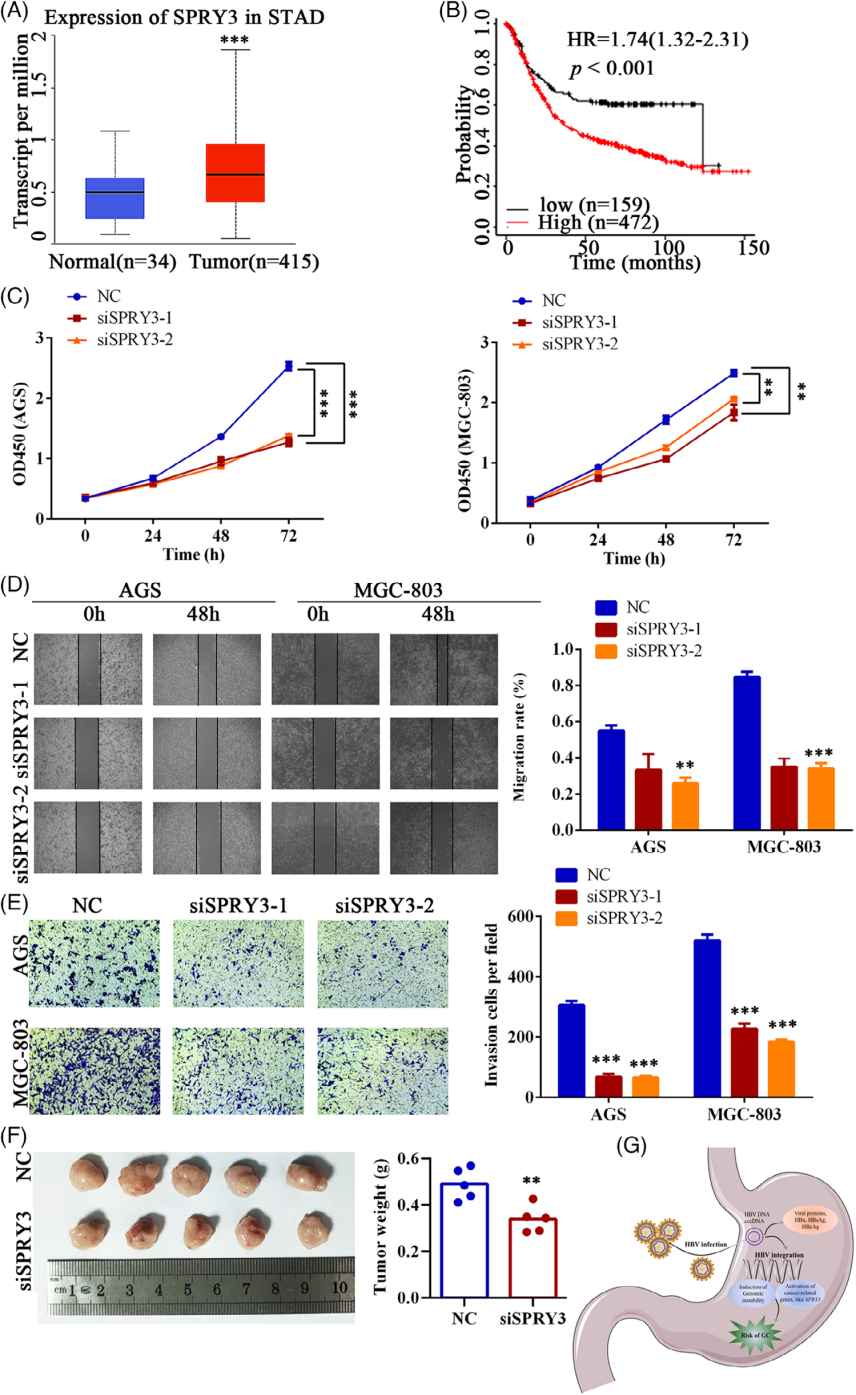
**Functional experiments of sprouty RTK signaling antagonist 3 (*SPRY3*) in gastric cancer**. (A) Expression analysis of the hepatitis B virus (HBV)‐targeted gene *SPRY3* in gastric cancer (GC) and non‐tumour tissues from The Cancer Genome Atlas (TCGA) database; (B) Kaplan‐Meier analysis of overall survival based on the expression level of *SPRY3* in GC patients from the TCGA database; (C) Relative cell growth in *SPRY3* knockdown AGS and MGC‐803 cells as measured by CCK8; (D) Migration assays of AGS and MGC‐803 cells transfected with si*SPRY3* or control; (E) Invasion assays of AGS and MGC‐803 cells transfected with si*SPRY3* or control; (F) Photographs and tumour weights of the subcutaneous tumours formed by *SPRY3* knockdown and control AGS cells (*n* = 5); (G) Graphical abstract in this study. *p < .05; **p < .01; ***p < .001.

Overall, the present study reveals the HBV infection and integration in GC by histological and molecular evidence. These findings provide a new direction for research into the mechanism of HBV‐associated GC initiation.

## AUTHOR CONTRIBUTIONS

Yifu He was responsible for the study concept and design, analysis and interpretation of data, critical revision of the manuscript for important intellectual content and obtaining funding. Hong Tu was responsible for the study design. Mengge Li and Shusheng Wu revised the manuscript. Mengge Li performed experiments, collected and analyzed data and wrote the manuscript. Ying Yan and Wenju Chen performed the literature search and carried out the data inclusion and extraction. Shusheng Wu and Huiqin Luo performed the quality assessment. Lulu Cao and Wenju Chen collected samples and analyzed data. Shusheng Wu and Jiayu Niu reviewed clinical data and performed statistical analyses. All authors read and approved the final manuscript.

## CONFLICT OF INTEREST STATEMENT

The authors declare no conflict of interest.

## FUNDING INFORMATION

This study was funded by grants from the National Natural Science Foundation of China (82203225 to Mengge Li), Fundamental Research Funds for the Central Universities (WK9110000172 to Mengge Li), Health Commission of Anhui Province Scientific Research Project (AHWJ2021b090 to Shusheng Wu and AHWJ2021b105 to Yifu He), Hefei Key Common Technology Research and Major Scientific and Technological Achievement Project (2021YL005 to Yifu He) and Anhui Province Key Research and Development Program Project (202104j07020044 to Yifu He).

## ETHICS STATEMENT

All human samples used in experiments were approved by the institutional ethics review committee of the First Affiliated Hospital of USTC and conducted according to the principles of the Declaration of Helsinki. Informed consent was obtained from all patients. We confirmed that all studies are conducted following relevant guidelines/regulations. All experiments involving mice were approved by the Animal Care and Use Committees of the First Affiliated Hospital of USTC, and were performed in accordance with the Institutional Animal Welfare Guidelines.

## Supporting information

Supporting Information

Supporting Information

## Data Availability

HBV capture sequencing data were uploaded to the NCBI database (accession number: PRJNA957723). The long‐read sequencing data were uploaded to the NCBI database (accession number: PRJNA956059). Other data supporting the findings in our study are available from the authors upon reasonable request.
