# Supplementary material for: Identification of hepatitis B virus infection and integration and its oncogenic role in gastric cancer
Source: Clin Transl Med. 2024 Mar 7;14(3):e1601. doi: 10.1002/ctm2.1601 (PMC10918734; doi:10.1002/ctm2.1601)
Supplement: Supplementary file 1 — Supporting Information [file CTM2-14-e1601-s001.docx]

**Supplementary Tables and Figures**

**Identification of HBV infection and integration and its oncogenic role in gastric cancer**

Mengge Li^1^, Shusheng Wu^1#^, Jiayu Niu^1^, Huiqin Luo^1^, Wenju Chen^1^, Lulu Cao^1^, Ying Yan^1^, Hong Tu^2^, Yifu He^1#^

**Supplementary tables**

**Table S1.** Characteristics of patients with gastric cancer in this study

| **Parameters** | **Gastric cancer patients**  **No. (%)** |
| --- | --- |
| Age | 64.8± 9.9 |
| Gender |  |
| Male | 9 (75.0%) |
| Female | 3 (25.0%) |
| Histologic grade |  |
| G1 | 1 (8.3%) |
| G2 | 1 (8.3%) |
| G3 | 10 (83.4%) |
| Pathological stage T |  |
| T1-2 | 2 (16.6%) |
| T3-4 | 10 (83.4%) |
| Lymph node metastasis |  |
| No | 3(24.6%) |
| Yes | 9(76.4%) |
| Tumor stage |  |
| I-II | 7 (58.3%) |
| III-IV | 5 (41.7%) |
| Subdivision |  |
| Cardia | 6 (50.0%) |
| Non-cardia | 6 (50.0%) |
| Lauren classification |  |
| Intestinal | 3(25.0%) |
| Diffuse | 7(58.3%) |
| Mixed-type | 2(16.7%) |
| Her-2 statue |  |
| Positive | 1 (8.3%) |
| Negative | 5 (41.7%) |
| Unknown | 6 (50.0%) |

**Table S2. HBV serum marker and DNA status in gastric cancer**

| **Sample**  **ID** | **Sex** | **Age** | **HBV serum marker** | | | | | **HBV DNA copies in serum (**IU/ml) | **HBV DNA in tissues** | |
| --- | --- | --- | --- | --- | --- | --- | --- | --- | --- | --- |
|  |  |  | **HBsAg** | **HBsAb** | **HBcAb** | **HBeAg** | **HBeAb** |  | **S gene** | **C gene** |
| 1T | female | 65 | + | - | + | - | + | Negative | + | + |
| 1N |  |  |  |  |  |  |  |  | + | + |
| 2T | male | 70 | + | - | + | + | - | 11900 | + | + |
| 2N |  |  |  |  |  |  |  |  | + | + |
| 3T | male | 53 | + | + | + | - | + | 3670 | + | + |
| 3N |  |  |  |  |  |  |  |  | + | + |
| 4T | female | 69 | + | - | + | - | + | ND | + | + |
| 4N |  |  |  |  |  |  |  |  | + | + |
| 5T | male | 69 | + | - | + | - | + | 2030 | + | + |
| 5N |  |  |  |  |  |  |  |  | + | + |
| 6T | male | 49 | + | + | + | - | + | 130 | + | + |
| 6N |  |  |  |  |  |  |  |  | + | + |
| 7T | male | 69 | + | - | + | - | + | Negative | - | - |
| 7N |  |  |  |  |  |  |  |  | + | - |
| 8T | male | 53 | + | - | + | - | + | Negative | - | - |
| 8N |  |  |  |  |  |  |  |  | - | - |
| 9T | male | 71 | + | - | + | - | + | 2050 | + | - |
| 9N |  |  |  |  |  |  |  |  | + | + |
| 10T | female | 80 | + | + | + | - | + | Negative | - | - |
| 10N |  |  |  |  |  |  |  |  | - | - |
| 11T | male | 55 | + | + | + | - | + | Negative | - | - |
| 11N |  |  |  |  |  |  |  |  | - | - |
| 12T | male | 75 | + | - | + | - | + | Negative | - | - |
| 12N |  |  |  |  |  |  |  |  | - | - |

HBV, hepatitis B virus; HBV DNA copies: negative, < 20IU/ml; ND, no detected.

**Table S4.** Category of HBV integration sites in the genome of gastric cancer tissues and the para-tumor tissues

| **Categories** | **GC tissues**  **No. (%)** | **Para-tumor tissues**  **No. (%)** | **Total**  **No. (%)** |
| --- | --- | --- | --- |
| Exon | 6 (3.43) | 7 (2.70) | 13 (3.00) |
| Intron | 72 (41.14) | 107 (41.31) | 179 (41.24) |
| UTR5 | 0 (0.00) | 1 (0.39) | 1 (0.23) |
| UTR3 | 3 (1.71) | 5 (1.93) | 8 (1.84) |
| Upstream (<1kb) | 1 (0.57) | 5 (1.93) | 6 (1.38) |
| Downstream (<1kb) | 0 (0.00) | 4 (1.54) | 4 (0.92) |
| Intergenic | 93 (53.14) | 130 (50.19) | 223 (51.38) |
| 1-10kb | 16 (9.14) | 17 (6.56) | 33 (7.60) |
| 10-50kb | 30 (17.14) | 42 (16.22) | 72 (16.59) |
| 50-100kb | 12 (6.86) | 26 (10.04) | 38 (8.76) |
| 100-200kb | 13 (7.43) | 14 (5.41) | 27 (6.22) |
| 200-300kb | 7 (4.00) | 4 (1.54) | 11 (2.53) |
| 300-400kb | 3 (1.71) | 3 (1.15) | 6 (1.38) |
| 400-500kb | 1 (0.57) | 4 (1.16) | 5 (1.15) |
| >500kb | 11 (6.29) | 20 (7.72) | 31 (7.14) |
| Total | 175 (100.00) | 259 (100.00) | 434 (100.00) |

GC: gastric cancer; UTR5: The 5′ untranslated region; UTR3: The 3' untranslated region.

**Table S5.** Recurrent HBV integration sites in gastric cancer patients.

| **Gene** | **Chromosome location** | **Insertion sites (distance, bp)** | **HBV location** | **Gene type** | **Gene function** | **Sample ID** |
| --- | --- | --- | --- | --- | --- | --- |
| *SPRY3* | chrY:59032246 | Intergenic  (68157) | 2317 | protein coding | Regulation of MAPK cascade | 4N |
|  | chrY:58978535 | Intergenic  (121868) | 537-671 |  |  | 2N |
|  | chrY:58989471 | Intergenic  (110932) | 1864-  1998 |  |  | 2T |
| *CHD6* | chr20:40247038 | Upstream | 2228 | protein coding | A core member of of chromatin remodeling complexes | 2N |
|  | chr20:40360823 | Intergenic  (113807) | 2904 |  |  | 2T |
| *CPNE4* | chr3:131806818 | Intergenic  (48368) | 2638 | protein coding | Membrane trafficking, mitogenesis and development | 2N |
|  | chr3:131254357 | Intronic | 1825 |  |  | 2T |
| *DPP10* | chr2:116342976 | Intronic | 573 | protein coding | Modulated the activity and gating characteristics of the potassium channel | 6T |
|  | chr2:117041886 | Intergenic  (438532) | 310 |  |  | 2N |
| *KLHL4* | chrX:86935296 | Intergenic  (10246) | 1029 | protein coding | Protein binding | 6T |
|  | chrX:86978740 | Intergenic  (53690) | 2626 |  |  | 2T |
| *MIR10524* | chr6:79017026 | Intergenic  (231954) | 2033-  2167 | MicroRNA | NA | 5N |
|  | chr6:79282034 | Intergenic  (295234) | 162 |  |  | 2N |
| *RBFOX1* | chr16:5896147 | Intergenic  (172878) | 2844 | protein coding | RNA-binding protein that regulates alternative splicing events | 2N |
|  | chr16:6247844 | Intronic | 2754 |  |  | 2T |
| *SLC6A15* | chr12:83786840 | Intergenic  (146430) | 370 | protein coding | Neuronal amino acid transport | 6T |
|  | chr12:85295958 | Intronic | 2141 |  |  | 2N |

NA: not available.

**Table S6. RT-qPCR primer sequences used in this study.**

| **Gene** |  | **Nucleotide sequence** |
| --- | --- | --- |
| ***SPRY3*** | Forward | 5-TGATGAGCCCTGCTCTTGTG-3 |
|  | Reverse | 5-TAGCCCTGTTGGCACAGATG-3 |
| ***GAPDH*** | Forward | 5-GGAAGCTTGTCATCAATGGAAATC-3 |
|  | Reverse | 5-TGATGACCCTTTTGGCTCCC-3 |

**Table S7. Logistics regression analyzed the relationship between *SPRY3* expression and clinicopathological features in TCGA gastric cancer data**

| **Characteristics** | **N** | **High** | **Low** | **Univariate analysis** | | **Multivariate analysis** | |
| --- | --- | --- | --- | --- | --- | --- | --- |
|  |  | **(N=117)** | **(N=126)** | ***p*** | **OR (95% CI)** | ***p*** | **OR (95% CI)** |
| Age (years) |  |  |  | 0.518 | 0.819(0.493-1.362) | 0.511 | 1.216(0.679-2.176) |
| <65 | 106 | 54 | 52 |  |  |  |  |
| ≥65 | 137 | 63 | 74 |  |  |  |  |
| Gender |  |  |  | **0.001** | **1.992(1.163-3.413)** | 0.086 | 1.674(0.929-3.015) |
| Female | 86 | 32 | 54 |  |  |  |  |
| Male | 157 | 85 | 72 |  |  |  |  |
| Lauren classification |  |  |  | 0.051 | 0.496(0.245-1.003) | **0.038** | **0.663(0.450-0.977)** |
| Diffuse | 48 | 27 | 21 |  |  |  |  |
| Intestinal | 100 | 53 | 47 |  |  |  |  |
| Unkown | 95 | 37 | 58 |  |  |  |  |
| Tumour stage |  |  |  | 0.064 | 1.537(0.921-2.563) | 0.742 | 0.874(0.393-1.945) |
| I-II | 112 | 48 | 64 |  |  |  |  |
| III-IV | 127 | 68 | 59 |  |  |  |  |
| Pathological stage |  |  |  | 0.366 | 1.154(0.650-2.046) | 0.537 | 1.247(0.618-2.517) |
| T1-2 | 64 | 29 | 35 |  |  |  |  |
| T3-4 | 178 | 87 | 91 |  |  |  |  |
| Lymph node metastasis |  |  |  | **0.024** | **1.874(1.104-3.182)** | 0.089 | 1.931 (0.905-4.118) |
| No | 91 | 35 | 56 |  |  |  |  |
| Yes | 152 | 82 | 70 |  |  |  |  |
| Distant metastasis |  |  |  | 1.000 | 1.075(0.365-3.168) | 0.890 | 0.919(0.919-3.040) |
| No | 220 | 106 | 114 |  |  |  |  |
| Yes | 14 | 7 | 7 |  |  |  |  |
| MSI_status |  |  |  | **0.000** | **0.244(0.111-0.539)** | **0.002** | **0.254(0.109-0.592)** |
| MSS/MSI-L | 202 | 108 | 94 |  |  |  |  |
| MSI-H | 41 | 9 | 32 |  |  |  |  |

**Table S8. Univariate and multivariate COX regression analysis of OS in 243 TCGA gastric cancer data**

| **Characteristics** | **Univariate analysis** | | **Multivariate analysis** | |
| --- | --- | --- | --- | --- |
|  | ***p*** | **OR (95% CI)** | ***p*** | **OR (95% CI)** |
| Age (years) | 0.06 | 1.768(0.975-3.206) | **0.001** | **3.206(1.608-6.393)** |
| ≥65 VS <65 |  |  |  |  |
| Gender | **0.048** | **1.966(1.006-3.839)** | **0.036** | **2.200(1.054-4.591)** |
| Male VS Female |  |  |  |  |
| *SPRY3* expression | **0.047** | **1.788(1.009-3.167)** | **0.038** | **1.321 (1.109-2.461)** |
| High VS Low |  |  |  |  |
| Lauren classification | 0.106 | 0.733(0.502-1.069) | 0.08 | 0.662(0.417-1.050) |
| Diffuse VS Intestinal VS Unkown |  |  |  |  |
| Tumour stage | **0.002** | **2.773(1.471-5.230)** | **0.033** | **3.148(1.098-9.020)** |
| III-IV VS I-II |  |  |  |  |
| Pathological stage T | 0.147 | 1.655(0.837-3.273) | 0.751 | 0.876(0.388-1.978) |
| T3-4 VS T1-2 |  |  |  |  |
| Lymph node metastasis | 0.191 | 1.501(0.817-2.759) | 0.193 | 0.546(0.219-1.359) |
| Yes VS No |  |  |  |  |
| Distant metastasis | **0.004** | **3.25(1.445-7.308)** | **0.001** | **5.041(1.859-13.666)** |
| Yes VS No |  |  |  |  |
| MSI_status | 0.057 | 0.405(0.16-1.026) | 0.091 | 0.409(0.145-1.154) |
| MSI-H VS MSS/MSI-L |  |  |  |  |

**Table S9. Sequences of siRNA that used in this study.**

| **Name** | **Nucleotide sequence** |
| --- | --- |
| **NC** | UUCUCCGAACGUGUCACGU TT |
| **siSPRY3-1** | AGCAUUGCCAGCUCAAUGUTT |
| **siSPRY3-2** | GCCAGCAUUACACCCUCACTT |

**Supplementary figures**

**Fig. S1.**
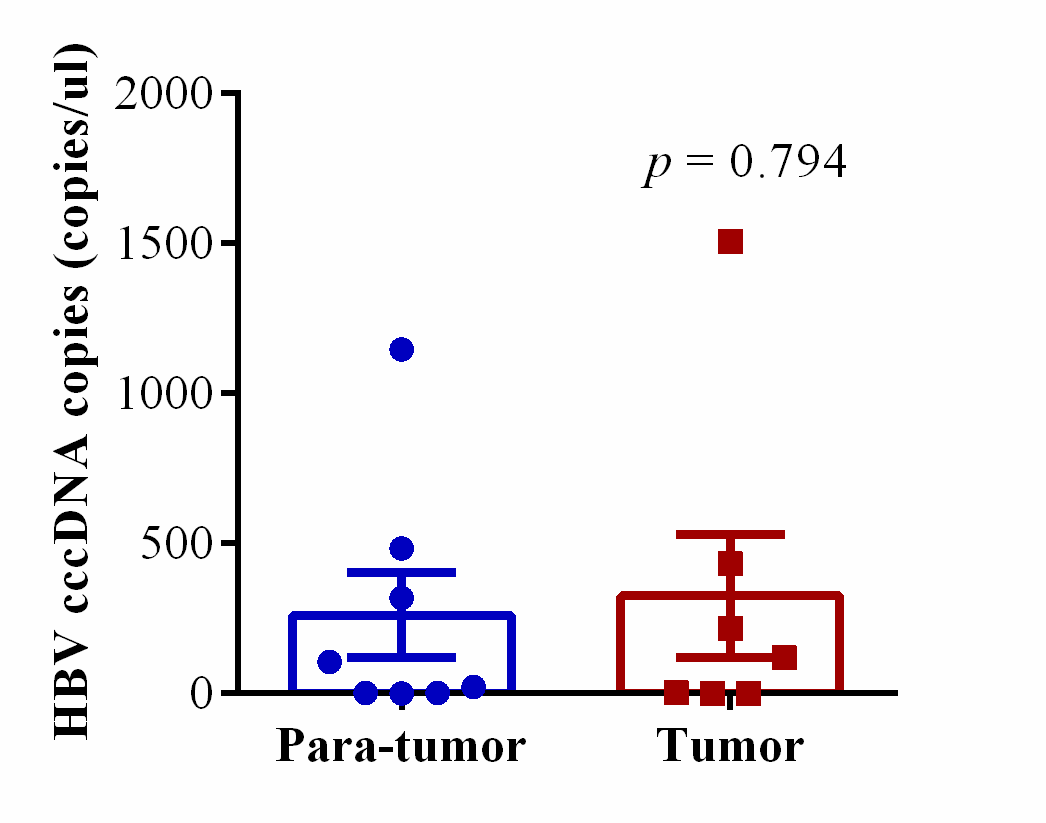
 The level of HBV cccDNA in the gastric cancer and para-tumor tissues by ddPCR.


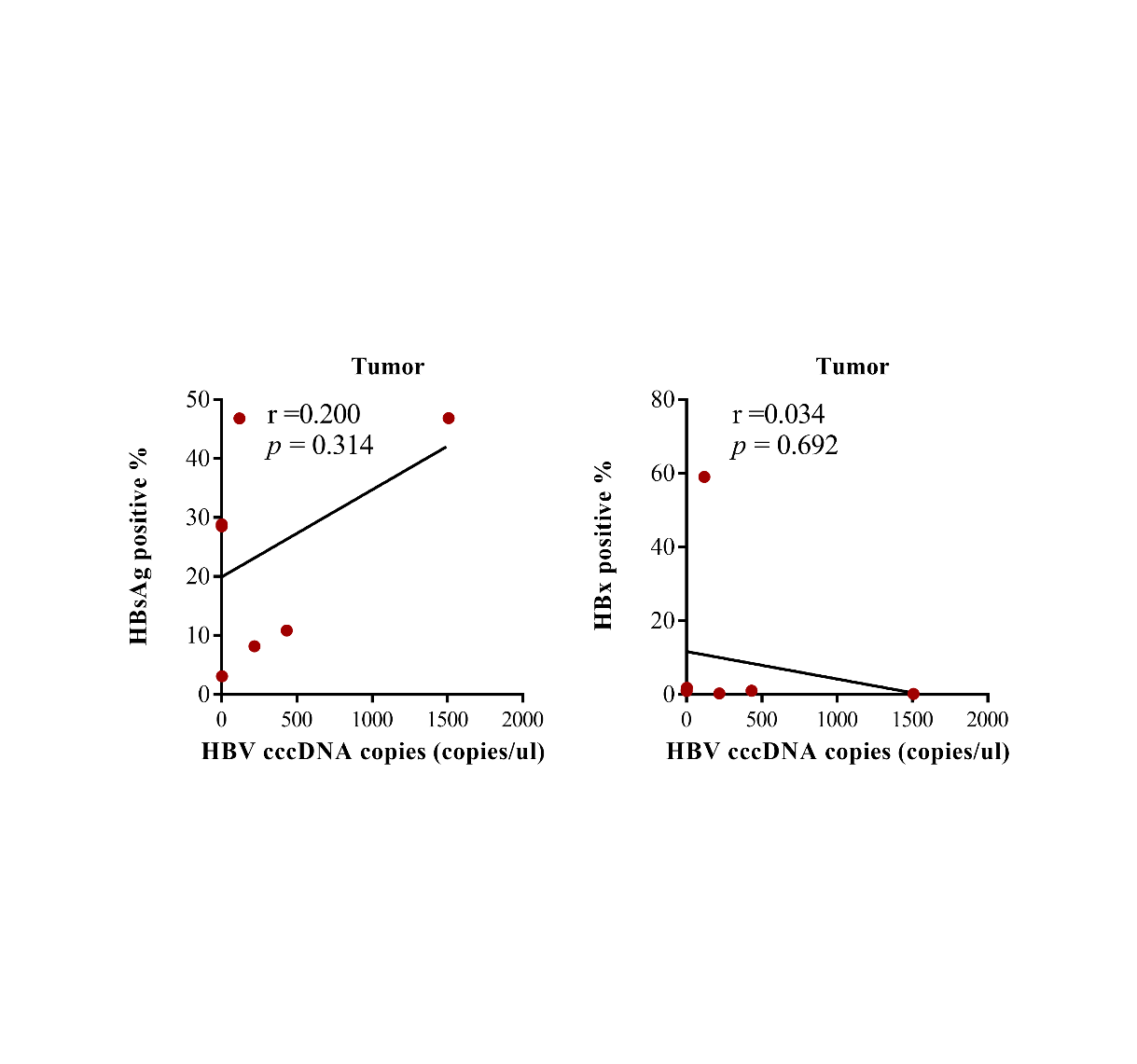


**Fig. S2.** Correlation analysis between viral proteins (HBsAg and HBx) and HBV cccDNA in gastric cancer tissues.

**
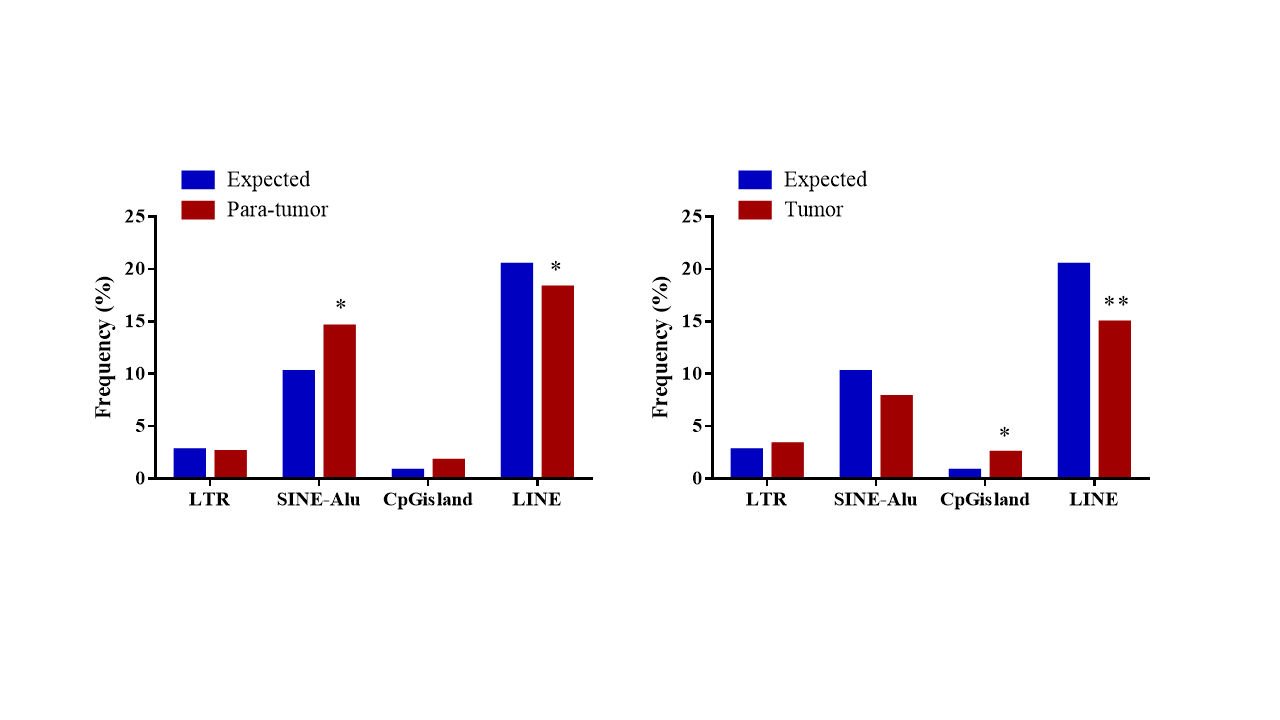
**

**Fig. S3.** Enrichment the genomic instability-related genomic elements in the flanking regions of the integrated sites in the para-tumor tissues (A) and gastric cancer tissues (B). LINE, long interspersed nuclear element; SINE-Alu, satellite and short interspersed nuclear element -Alu repeats; LTR, long terminal repeat. *, *p* < 0.05; **, *p* < 0.01.

**
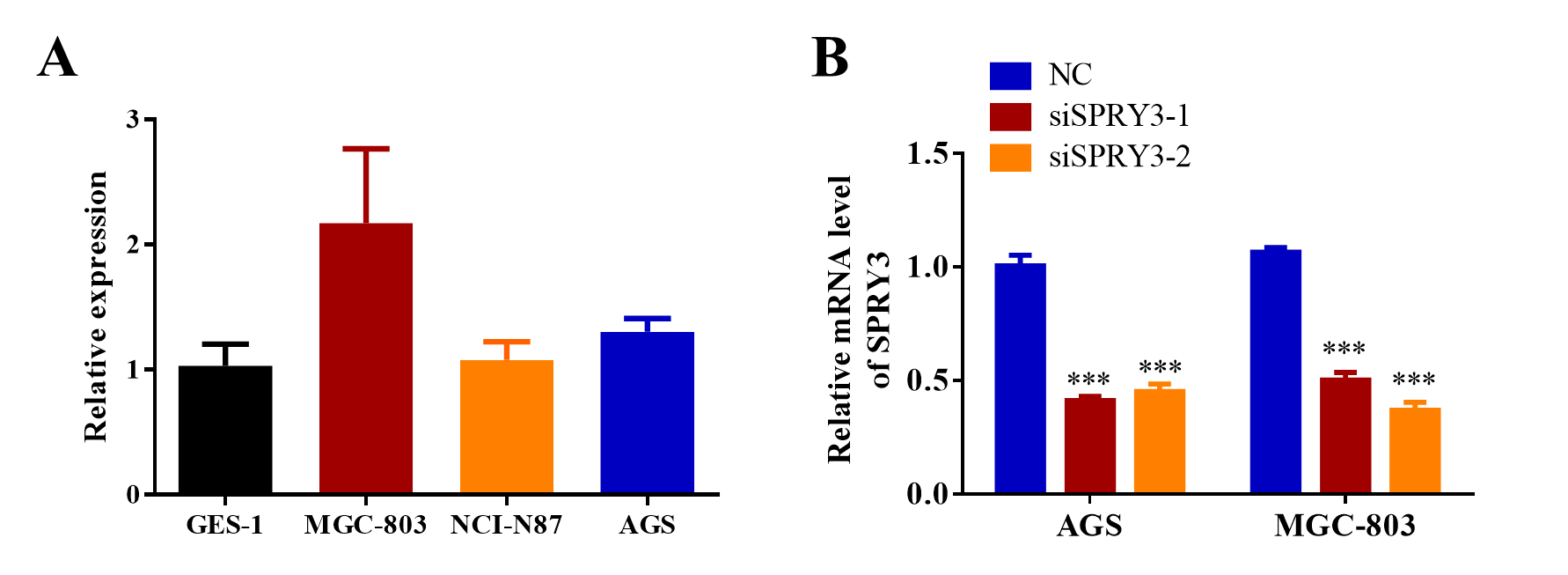
**

**Fig. S4.** Relative mRNA level of *SPRY3* in the human normal gastric epithelial cell line (GES-1) and GC cell lines. (A). Relative mRNA level of *SPRY3* in GSE-1 and GC cells (MGC-803, NCI-N87 and AGS); (B). Relative mRNA level of *SPRY3* in GC cells (AGS and MGC-803) infected with si*SPRY3* or control. *, *p* < 0.05; **, *p* < 0.01; ***, *p* < 0.001.
